# Evaluation and Improvement of Bio-Based Sustainable Resin Derived from Formic-Acid-Modified Epoxidized Soybean Oil for Packaging Applications

**DOI:** 10.3390/polym15214255

**Published:** 2023-10-29

**Authors:** Abdus Sobhan, Shahab Saedi, Magdalene Hoff, Yaohua Liang, Kasiviswanathan Muthukumarappan

**Affiliations:** Department of Agricultural and Biosystems Engineering, South Dakota State University, Brookings, SD 57007, USA; abdus.sobhan@jacks.sdstate.edu (A.S.);

**Keywords:** green epoxidation, formic acid, resin film, resin polymer, soybean oil

## Abstract

Bio-based epoxy resin materials have obtained significant attention in the packaging industry due to concerns about the environmental and economic impacts of traditional petroleum-based plastics. The aim of this research is to improve bio-based resins’ properties by investigating varying formic acid contents in the presence of a green catalyst and characterizing their physical, chemical, and mechanical properties for further scaled-up bio-based resin production for industrial packaging applications. The crude soybean oil was epoxidized with formic acid as an oxidizing agent at varying equivalent weights of 10:1 to 10:10 of soybean oil: formic acid in the presence of hydrogen peroxide and choline chloride-oxalic acid as a bi-functional green catalyst. The effect of increasing the amount of formic acid used to epoxidize crude soybean oil was evaluated with infrared (IR) spectroscopy, rheological, and epoxy yield measurements. The results demonstrated that formic acid significantly influenced the epoxidation of soybean oil, leading to a higher conversion of carbon-carbon double bonds, with a selectivity of 98% when the ratio of soybean oil to formic acid was between 10:5 and 10:10. The bio-resin film was formulated using the improved epoxidized soybean oils—from ESO (10:2.5) to ESO (10:10)—and equal amounts of acrylic acid. The results showed that resin films led to an improvement in tensile strength (ca. 180 MPa) and thermal stability at 360 °C. Although further research is necessary, this study provides valuable insights for designing an effective epoxidation process for renewable sources and developing bio-resin materials for future packaging applications.

## 1. Introduction

Packaging plastic pollution has emerged as a critical global environmental concern, with more than 90% of packaging plastics, including items such as disposable straws, tableware, and bags, being derived from petroleum-based plastic polymers [1]. These take up space in landfills and lead to the accumulation of plastic waste, which contributes to soil fertility reduction and ecosystem destruction. To address this issue, there has been extensive research and exploration conducted into the development of sustainable bio-based resins sourced from renewable biomaterials, including plant-based oils, natural fibers, and carbohydrates. These bio-based resins offer significant potential for reducing carbon emissions and minimizing environmental impact throughout their life cycle [2,3,4], both as natural fibers [2] and carbohydrates [5]. They have the potential to lower carbon releases and minimize environmental impact at the end of their life cycles [6]. Therefore, by utilizing renewable sources, these resins have the capacity to contribute to sustainability efforts and combat the detrimental effects of existing plastic packaging pollution [7,8]. 

The starting materials for petroleum-based plastic polymers are epichlorohydrin and bisphenol A, and petroleum-based plastic polymers are derived from petroleum or non-renewable resources [9]. Bisphenol A has also been identified as an endocrine disruptor, disrupting the hormonal system of humans and wildlife [10]. To reduce dependence on non-renewable or plastic-based sources, plant-based source materials are widely sought and in growing demand in the market. Out of all plant-based sources, soybean oil stands out as the most widely used oil because of its mass production capabilities, cost-effectiveness, and easy conversion to biopolymer via the ring opening of tertiary oxirane groups [11]. Most of the fatty acids contained in soybean oil are unsaturated, with a notable proportion of linoleic acid; these are additionally complemented by palmitic acid, oleic acid, stearic acid, and linolenic acid [12]. Unsaturated fatty acids are susceptible to epoxidation and bio-resin development.

Epoxidized soybean oil (ESO) is a bio-based product obtained by converting the carbon-carbon double bonds of soybean oil into epoxy groups; it is more chemically reactive compared to soybean oil [13]. Meanwhile, its unique properties have attracted considerable attention, particularly in the field of green epoxy resin for developing both monomer and polymer resin [14]. The epoxidation process of soybean oil involves the use of hydrogen peroxide as an oxygen donor, with formic acid serving as an oxygen carrier. Subsequently, this performic or peracetic acid reacts with the unsaturated bonds present in vegetable oil, resulting in the production of epoxide groups, also known as oxirane oxygen content (OOC). These epoxide groups are essential for crosslinking processes, which involve the formation of chemical bonds between molecules [5,15]. Moreover, epoxidized soybean oil possesses substantial potential for diverse industrial applications. Its versatility extends to areas such as plasticizers, adhesives, sealants, coatings, the formulation of epoxidized biodiesel, paint manufacturing, and the development of resins [16].

Formic acid is a preferred catalyst for the green epoxidation of soybean oil because it is a relatively mild acid that can efficiently promote the reaction while avoiding side reactions that can lead to undesirable byproducts [4]. Additionally, formic acid is readily available and inexpensive, making it an attractive choice for large-scale industrial applications. The reaction is typically conducted in the presence of an oxidizing agent such as hydrogen peroxide or peracetic acid, which provides the oxygen molecule needed for the epoxidation [12]. Specifically, formic acid functions as a proton donor that initiates the chemical reaction between the peroxide groups and unsaturated double bonds remaining in the soybean oil [4]. In terms of epoxidizing, various epoxidation methods have been employed, including heterogeneous catalytic systems utilizing acidic ion exchange resins, epoxidation via phase-transfer catalysts, and chemoenzymatic epoxidation [17]. Notably, formic-acid-modified epoxidation in terms of OOC (Oxygen Oxirane Content) or EEW (Epoxy Equivalent Weight) has the potential to yield higher OOC and lower EEW values due to its pronounced selectivity, eco-friendliness, and high degree of purity [18]. The role of formic acid as a catalyst in this reaction is to activate the peroxide, which is the oxidizing agent used in the epoxidation reaction [19]. Based on the existing literature, there is a lack of research on the enhancement of bio-based resins tailored for applications in the bio-based plastic packaging industry. The present study focuses on addressing this gap by developing a method to modify epoxidized soybean oil (ESO) using various formic acid contents and their derivatives, to attain desired properties in the resin for industrial packaging applications. This modification process involves utilizing a deep eutectic solvent, specifically choline chloride-oxalic acid, to dissolve and condense both epoxy monomers. This research is also a continuation of a previous study that examined the utilization of deep eutectic solvents for epoxidized soybean oil and characterized their potential for bio-resin development [20]. Hence, this research aims to achieve the following objectives: (1) to verify and improve epoxy yields in epoxidized soybean oil derived from crude soybean oil, by adjusting formic acid contents and assessing the improved functional properties of the bio-resin, and (2) to fabricate cyclic epoxy resin film strips using the derived epoxidized soybean oil and evaluate their mechanical and thermal properties for sustainable applications.

## 2. Materials and Methods

### 2.1. Materials

Refined soybean oil with an iodine value of 130.2 g (I_2_/100 g) was supplied by Commercial and Trading Co., Ltd. located in Volga, SD, USA. Oxalic acid dihydrate and choline chloride, both with a purity of 99%, were all purchased from Thermo Scientific™ (Waltham, ME, USA). Wijs solution, sodium thiosulfate (99%), potassium iodide (99%), and deuterated chloroform containing 0.03% tetra-methyl silane (TMS) were obtained from Sigma-Aldrich (St. Louis, MO, USA). Hydrogen peroxide (50%) and formic acid (99%) were supplied by Thermo Scientific™ (Waltham, ME, USA). 

### 2.2. Preparation and Epoxidation of Refined Soybean Oil

**Figure 1:**
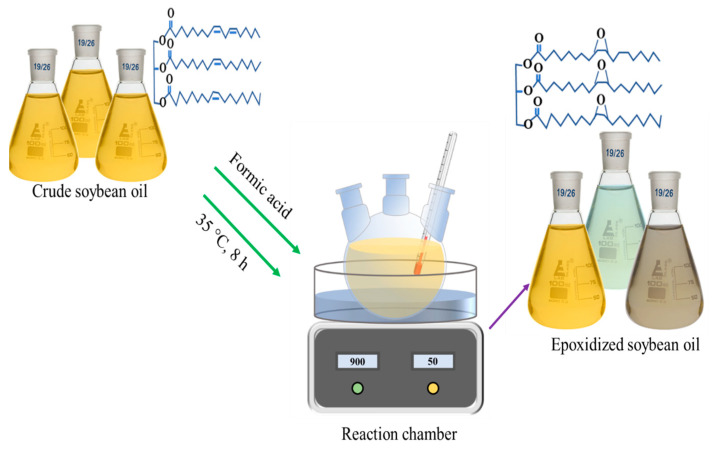
Synthesis approach of epoxidations of refined crude soybean oil reacting with hydrogen peroxide, formic acid, and deep eutectic solvent.

A deep eutectic solvent (DES) of choline chloride with oxalic acid was prepared according to a previously developed method [21]. In brief, oxalic acid dihydrate (OA) was first blended with dried choline chloride (ChCl) in a molar ratio of 1:1. Next, the mixture of choline chloride and oxalic acid was heated and stirred in an oil bath at a temperature of 100 °C to ensure a thorough mixing of the two components. Then, the resulting choline chloride with oxalic acid solvent was stored in a refrigerator after cooling to room temperature. The epoxidation of soybean oil involved a reaction between the soybean oil and performic acid, which was yielded in situ from the reaction of hydrogen peroxide with formic acid. In brief, 10 g of soybean oil and 0.5 g of DES were placed into a three-neck round beaker prepared with a reflux condenser, a mechanical stirrer, and a thermometer. The equivalent weight of formic acid, ranging from 10:1 to 10:10, was dropped into the mixture in the beaker; for example, 1 g of formic acid was dropped into 10 g of soybean oil for a ratio of 10:1, while 10 g of formic acid was added to 10 g of soybean oil for a ratio of 10:10. Then, 20 g of hydrogen peroxide was gradually added using a continual dropping funnel over a period of 15 min to avoid excessive heating of the equipment. Throughout the epoxidation reaction, the reaction temperature was kept between 40 and 45 °C in an incubator under continual stirring for 6 h. After completion of the designated reaction time, the resulting mixture was cooled to room temperature and subjected to centrifugation to separate unreacted formic acid. Next, the separated resultant mixture was further washed 3 times with deionized water and 1 time over anhydrous sodium sulfate, through centrifugation for at least 10 min. Lastly, the remaining washed oil product was acquired and named as epoxidized soybean oil (ESO). The derived ESOs samples were renamed depending on the equivalent weight of formic acid, as 10:1, 10:2.5; 10:5, 10:7.5, and 10:10 ESO. A schematic diagram of synthesis approach of epoxidations is shown in Figure 1.

### 2.3. Physicochemical Analysis of Derived Epoxidized Soybean Oil (ESO) 

#### 2.3.1. Determination of Iodine Value and Oxygen Content in ESO

Iodine value is a measurement used to determine the degree of unsaturation in an oil or fat, while the oxirane number is a measure that indicates the amount of epoxide groups in an oil or fat. In this study, the iodine values of the derived ESO samples were determined using a Wijs solution, as described in a previous study [15,18]. The oxirane number of the ESO samples was determined through potentiometric titration, as described in a previous study [7].

The selectivity and conversion of double bonds of epoxidized soybean oil were measured as follows:

(1) $$\mathrm{Selectivity}=\frac{[Oxirane\;number]\times 253.8}{[Iodine\;number]soybean\;oil-[Iodine\;number]sample\times 16}$$

(2) $$\mathrm{Conversion}=\frac{(Iodine\;number)soybean\;oil-(Iodine\;number)sample}{(Iodine\;number)sobean\;oil}\times 100$$

#### 2.3.2. Determination of Viscosity

The viscosities of the different ESO samples were measured using the previously reported method with a slight modification [22]. Prior to determination of the viscosity, the rheometer (ATS Rheosystems, Bordentown, NJ, USA) was first calibrated according to the manufacturer’s instructions and equilibrated to room temperature. The epoxidized sample was loaded onto the rheometer cup and then the viscometer spindle was put into the cup to record the resulting shear stress or strain rate and determine the sample’s viscosity. The viscosity analysis of each ESO sample was determined in triplicate. 

#### 2.3.3. Fourier-Transform Infrared Spectroscopy (FTIR)

The FTIR spectra of the various ESO samples were examined using a Tensor 37 spectrophotometer (Thermo Fisher Scientific, Madison, WI, USA). The spectra were acquired from 500 to 4000 cm^−1^, with 64 scans performed and a resolution set at 4.0 cm^−1^.

### 2.4. Preparation of Acrylate Epoxy Resin Thermoset Film 

To prepare the acrylate epoxy thermoset resin films and confirm their thermal and mechanical properties, 2 g of derived ESO samples 10:1 to 10:10 was mixed with 2 g of acrylic acid (AC). The resulting mixture solution was then mildly stirred for 15 min at a temperature of 30 °C, to generate an acrylate-epoxidized soybean oil suspension. Subsequently, each suspension was separately molded and subjected to a controlled temperature ramp in an oven, involving stages of 90 °C for 2 h, 100 °C for 2 h, and 110 °C for 4 h for film casting and to initiate chemical bindings between the ESO and acrylic acid. Next, the dried films were subjected to an additional annealing step at 120 °C for 4 h, to facilitate film casting. The film samples were put in a desiccator with a relative humidity of 57–60% (achieved using saturated sodium bromide) and left at room temperature for 72 h. Next, the dried epoxy films were delicately peeled off from the molds. Prior to any further analysis or characterization, the films were conditioned in a container at room temperature and a relative humidity of 50–60% for 2 days.

### 2.5. Differential Scanning Calorimeter 

The thermal properties of the films were measured using a differential scanning calorimeter (DSC) (DSC-Q2000 TA Instruments, New Castle, DE, USA). To prepare the samples for DSC analysis, we followed a formerly reported method with a minor modification [20]. In brief, 3 mg of each film sample were weighed and put into a sealed pan, using a lid to ensure that the samples were securely contained within the pan during the analysis. [23]. Next, the sample specimens were subjected to heating in a nitrogen atmosphere within the temperature range of 20 to 400 °C at a heating rate of 10 °C/min, while maintaining a gas flow rate of 20 mL/min. A full aluminum empty pan was used as a reference.

### 2.6. Tensile Analysis (TA) 

A Tensile Analyzer Tester (Texture Technologies Corp., Scarsdale, NY, USA) was used to assess the mechanical properties of the developed film samples following our prior-developed method [24,25]. For analysis, film strips (50 × 20 mm) were held in the grip of the texture analyzer, which was connected to Texture Exponent 32 software. The crosshead grip speed was between 45 and 50 mm/min, and a distance of 45 to 50 mm was maintained between the grips.

(3) $$\mathrm{Tensile}\;\mathrm{strength}=\frac{Tensile\;Force\;(N)}{Area\;(sq.m)}$$

(4) $$\mathrm{Strain}=\frac{Deformation}{Original\;length}$$

### 2.7. Analysis of Swelling Degree and Water Solubility of the Resin Films

In this study, the swelling degrees and water solubilities of the resin films were determined by following the previously developed method with minor modifications [26,27]. For this, dry film samples—measuring 50 by 20 mm in size and with thicknesses ranging from 0.15 to 0.18 mm—were placed into distilled water for a duration of 24 h. After 24 h, the undissolved parts of the film samples were collected and subsequently dried in an oven at 105 °C. The calculations for swelling degree and water solubility were then performed as follows:

Swelling degree = (M_1_ − M_0_)/M_0_ (5)

Water solubility (%) = (M_0_ − M_2_)/M_0_ × 100 (6)

where *M*_0_ is the initial weight of the m, *M*_1_ indicates the weight of the wet film sample, and *M_2_* represents the dry weight of the undissolved film sample.

### 2.8. Opacity

The opacity measurements of the bio-resin film were assessed with a spectrophotometer (Agilent Technologies, Santa Clara, CA, USA). The light absorbance of film specimens (which had dimensions of 10 × 35 mm and thicknesses ranging from 0.15 to 0.18 mm) was measured at a wavelength of 600 nm. An empty cuvette was used as the control, and the opacity was determined via

Opacity (mm^−1^) = A_600_/T (7)

A_600_ denotes the light absorbance in the films at 600 nm, and *T* indicates the film thickness (mm).

## 3. Results and Discussion

### 3.1. Properties of Chemically Modified Epoxidized Soybean Oil (ESO)

#### 3.1.1. Evolved Iodine Values, Conversion Rate, Selectivity, and Oxirane Levels of ESO Samples

**Figure 2:**
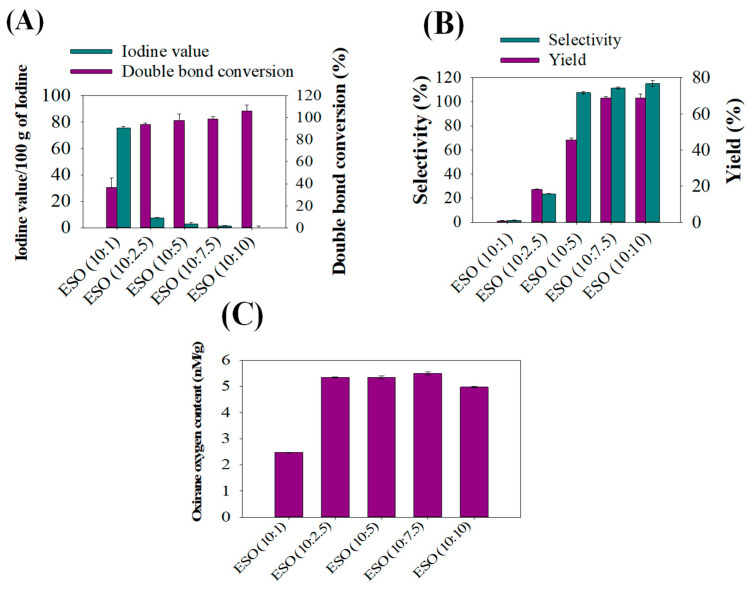
Effects of varying levels of formic acid on the epoxidation of refined soybean oil: (**A**) iodine value versus double bond conversions, (**B**) selectivity of the epoxidation reaction versus epoxy yield, and (**C**) oxirane oxygen contents determined in various epoxidized soybean oils (ESOs).

To analyze the effectiveness of formic acid in improving epoxy bio-resins during soybean oil epoxidation, the iodine value, double bond conversion, selectivity, epoxy yields, and Oxirane Oxygen Content (OOC) profiles were determined. As can be seen in Figure 2A, the iodine value found in ESO (10:1) was 75/g and decreased to 1/g in ESO (10:10) during epoxidation. This phenomenon of decreasing iodine values with increasing formic acid is related to the fact that during epoxidation, double bonds of soybean oil were dramatically transformed with increasing formic acid amounts during epoxidation. In addition, formic acid is an electron acceptor, so increasing formic acid contents effectively accepts oxygen released by hydrogen peroxide in reactions and forms perchloric acid to epoxidize soybean oil samples perfectly during reactions [12]. It has been seen that changing the equivalent weight of formic acid during epoxidation greatly influenced iodine values, and substantially lower iodine values were obtained in ESO (10:10) samples. It was noted that the iodine number represents the number of double bonds; therefore, the iodine number decrease indicates the conversion of unsaturated double bonds of soybean oil into saturated single bonds. Figure 2B shows comparisons of selectivity with epoxy yields, and an increasing epoxy yield with selectivity was noticed with increasing formic acid content at equivalent weights of 10:1 to 10:10 of soybean oil and formic acid. In contrast, samples epoxidized with a 10:1 ratio of soybean oil and formic acid exhibited significantly lower epoxy yields with lower selectivity. This phenomenon is attributable to double bond conversions or the ring opening of some of the epoxide groups, because the interaction between O2^−^ and H^+^ molecules of hydrogen peroxide and formic acid during epoxidation resulted in the formation of hydroxy formic acid. This interaction also involves the donation of electrons during double bond conversions [28]. Figure 2C presents OOC values of different ESO samples, ranging from 10:1 to 10:10. As can be inspected, increasing levels of OOC were found with increasing contents of formic acid at 10:2.5, which confirmed that formic acid has significantly increased the epoxidation rate of soybean oil. However, there is no significant difference in OOCs between 10:2.5 and 10:7.5 ESO, and OOC values decreased to 5 nM/g for ESO (10:10).

#### 3.1.2. Viscosity and pH of Epoxidized Soybean Oil Samples

**Figure 3:**
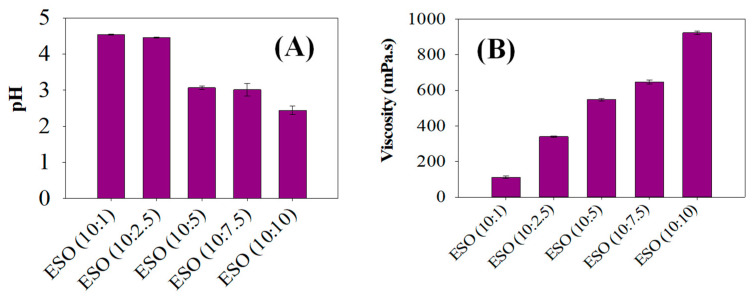
(**A**) Viscosity analysis of various epoxidized soybean samples and (**B**) pH levels of various epoxidized soybean samples between ESO (10:1) and ESO (10:10).

The pH and viscosity values of various ESO samples which were epoxidized with different equivalent weights of formic acid were determined, as seen in Figure 3. It is seen that the pHs of ESO (10:1) and ESO (10:2.5) were comparatively higher, leading to pHs of over 4.5. However, the pH was further reduced between ESO (10:5) and ESO (10:10), resulting in a pH of 3. The decreasing pH values could be caused by the release of hydrogen bonds from the formic acid in the epoxidized solution. Formic acid is recognized for bridging the gap between the aqueous and organic phases, and the acidic protons within formic acid contribute to this reduction in pH value [28,29]. These acidic protons released from formic acid during soybean oil epoxidations were responsible for the decreasing pHs of the ESO samples. This was because the extent of hydrogen bonding led to the greater release of acidic protons, bringing about the decrease in pH.

As can be seen in Figure 3B, the viscosity increased as the equivalent weights of formic acid content increased in ESOs with 10:1 to 10:10 of soybean oil: formic acid ratios. The increased viscosity of the ESOs was sequentially seen between ESO (10:1) and ESO (10:10), resulting in a higher viscosity of 950 mPa.s in ESO (10:10). This phenomenon of increasing viscosity values could be caused by the stronger van der Waals forces with the lengthening alkyl chain in the ESO samples [30]. The higher viscosity of ESO can be attributed to the presence of the epoxide groups. These groups are polar and tend to interact with each other, leading to increased intermolecular forces and thus higher viscosity [20]. Additionally, the epoxide groups can form hydrogen bonds with other molecules, which further contributes to the higher viscosity. A higher degree of epoxidation results in a higher concentration of epoxide groups, which in turn leads to a higher viscosity. The epoxidation process and the resulting formation of epoxide groups are the main reasons for the higher viscosity observed in epoxidized soybean oil.

#### 3.1.3. Fourier-Transform Infrared (FTIR) Analysis

**Figure 4:**
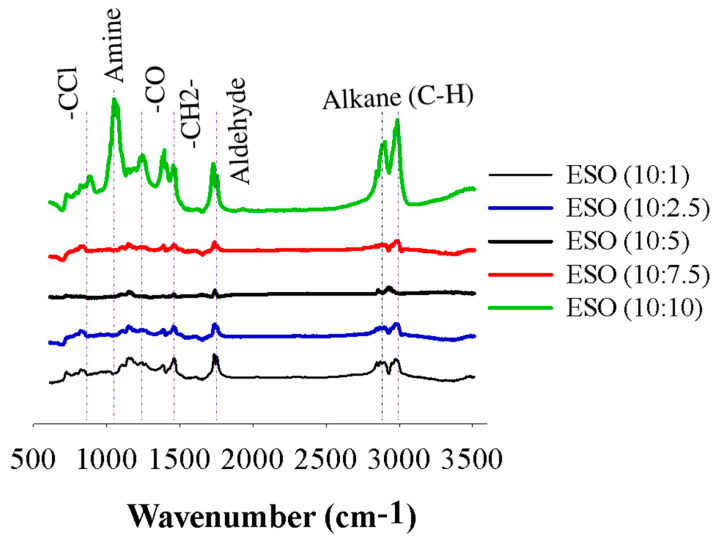
FTIR spectra of epoxidized soybean oil samples of ESO (10:1) to ESO (10:10).

FTIR analysis is a common technique used to identify functional groups in organic compounds, including epoxidized soybean oil (ESO). The IR bands for each individual ESO sample, ranging from ESO (10:1) to ESO (10:10), were determined to confirm the functional groups produced in the ESO samples (Figure 4). The broad peak between 2995 and 2829 cm^−1^ of the aliphatic alkanes (C−H) stretching was displayed. The characteristic peak of the aldehyde (−CHO stretch) occurred in the range of 1730 to 1780 cm^−1^ [31]. This band that occurs at the same wavelength suggests its association with the −CH2 stretching vibration. Additionally, the band at 1460 cm^−1^ can be attributed to the stretching vibration of carbon-carbon single bonds (−C−O−C) connected to epoxy groups in the ESO samples. Notably, when soybean oil was epoxidized using a 10:10 ratio of formic acid as an initiator, the intensity of bands related to the epoxide groups significantly increased. This observation strengthens the hypothesis that the predominant reaction in the presence of higher concentrations of formic acid is the ring opening of the epoxy groups. The presence of stretching bands was identified at 1270 cm^−1^ and attributed to the asymmetric stretching vibration of amines. Another band at 1400 cm^−1^ shows a broad signal and is associated with the stretching vibration of C=C double bonds conjugated with epoxidized samples. Additionally, the appearance of a distinctive −CCl stretching band at 700 cm^−1^ confirms the presence of carbon-chloride groups in the epoxidized samples [1]. On the one hand, no bands were observed between 3000 and 4000 cm^−1^. Overall, the peak intensity for the epoxidized soybean oil using a 10:10 ratio of formic acid was comparatively higher compared to the other samples. 

### 3.2. Physical Property Analysis of Epoxidized Bio-Resin (EBR) Films

#### 3.2.1. Thermal Assessment of EBR Films

**Figure 5:**
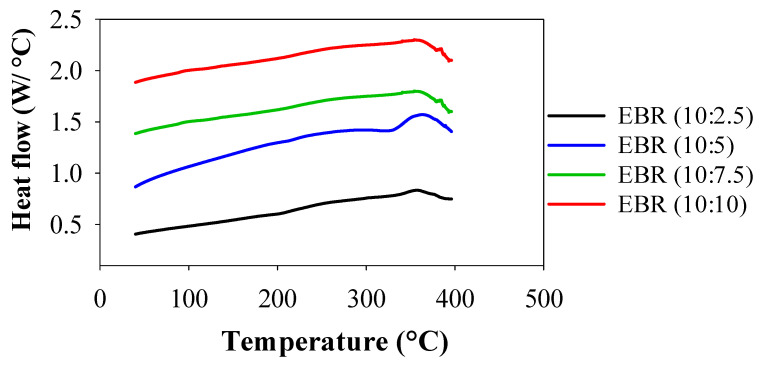
Comparison of thermal studies of the bio-resin films between EBR (10:2.5) and EBR (10:10).

DSC was used to assess the thermal degradations and behaviors of EBR films, and the resulting thermograms are shown in Figure 5. Between 20 and 400 °C, consecutive endothermic and exothermal DSC peaks were seen for the films of EBR (10:5), and one exothermal peak appeared for EBR (10:2.5), EBR (10:7.5), and EBR (10:10). The first endothermic peak for EBR (10:5) film appeared between 321 and 334 °C, indicating the evaporation of bound water. However, the peak shifted and occurred at 360.30 °C for all the films, indicating the higher thermal stability of EBR films. This second exothermic peak, called crystallization (Tc), was seen in all films at 360.30 °C. No thermal conversion peaks were observed in the EBR films prior to 321 °C. This indicates that the temperature inflection points catalyzed by higher amounts of formic acid were significantly higher than 321 °C. In addition, it was concluded that the large amounts of epoxy resin in epoxidized soybean influenced the increased thermal stabilities, indicating that the EBR films are more thermally stable due to the higher conversion of double bonds during the catalysis of soybean oil [32]. After that, for all films, it began to decompose and release exothermic heat flow from 360.30 °C, indicating that thermal degradation of the films occurred from this temperature of 360.30 °C. The temperature at which the thermal conversion peak occurred has a significant relationship with thermal stability and indicates the material’s ability to withstand higher temperatures, offering valuable insights for its practical use in polymer applications [10]. The results for thermal stability suggested that the epoxidized film exhibited outstanding resistance to high temperatures, making it a promising candidate as an engineering material for industrial applications.

**Figure 6:**
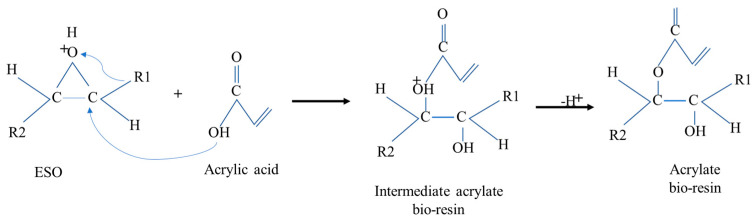
Mechanisms of formation of soybean bio-resin film (acrylate).

The acrylation of epoxidized soybean oil with acrylic acid occurred during casting, to develop EBR films through a chemical and thermal modification that inserted acrylate groups into the epoxidized bio-resin film’s structure. As shown in Figure 6, acrylate groups contain a double bond, which is often used to enhance the properties of resins, such as their thermal, mechanical, and cross-linking abilities, making the resin suitable for various applications.

#### 3.2.2. Mechanical Analysis of Epoxidized Bio-Resin Films

**Figure 7:**
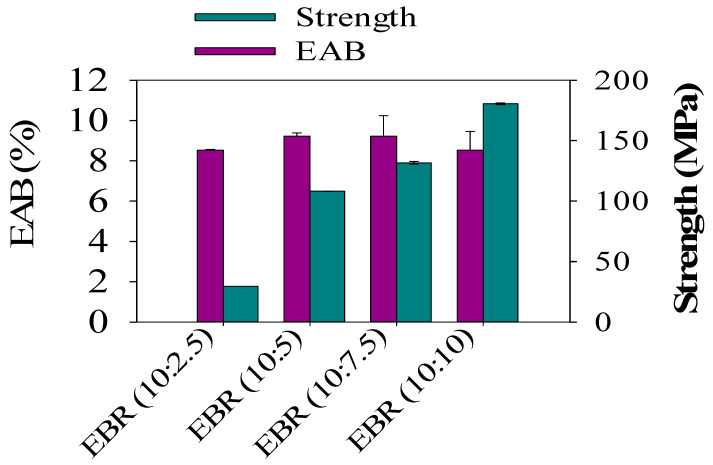
Tensile strength and elongation at break of the epoxidized bio-resin (EBR) films.

In order to analyze the mechanical properties of the developed bio-resin films, which were made from different ESO contents and acrylic acid, different film formulations for the epoxidized soybean resin films from EBR (10:2.5) to EBR (10:10) were investigated. The tensile strength (TS) of the EBR films sharply increased from 6.80 to 170 MPa when formic acid contents in the epoxidized soybean oil ranging from ESO (10:2.5) to ESO (10:7.5) were introduced. As can be demonstrated in Figure 7, EBR (10:10) exhibited a significant tensile strength (TS) of 170 MPa, along with an elongation at break (EAB) of 7%. This result can be attributed to the notable increase in epoxy yields achieved by the increasing formic acid contents during the epoxidation process [1]. TS was minimal for the film with EBR (10:2.5) (*p* < 0.05), indicating a poor interaction between ESO and acrylic acid, which is consistent with the rough and heterogeneous cross-section observed in the tensile strength analysis. It was expected that the elongation at break (EAB) of the film would exhibit an opposite trend compared to the tensile strength (TS). However, no significant impact on the EAB of the films was observed from EBR (10:5) to EBR (10:7.5). This phenomenon can be attributed to the films undergoing different levels of deformation before breaking.

#### 3.2.3. Opacity and Water Resistance

Table 1 presents the evaluations of opacity and water resistance properties, to assess the integrity of the film samples in an aqueous solution. The opacity of a film is closely associated to its microstructure, where a higher opacity indicates lower transparency. In the case of EBR (10:10), the opacity was measured as 0.229 mm^−1^ and primarily attributed to the refractive index difference between ESO and acrylic acid. However, the opacity ranged between 0.122 and 0.173 mm^−1^ for other samples. This significant decrease indicates improved transfer properties between EBR (10:2.5) and EBR (10:7.5), resulting in enhanced light transmittance. Moreover, the opacity increased with higher contents of formic acid, possibly due to the heterogeneous structure of the bio-resin film, which hinders light transmittance and causes light dispersion [33]. Additionally, water resistance properties were evaluated to determine the film’s integrity in an aqueous environment. As indicated in Table 1, the swelling degree and water solubility were measured and studied to assess the water resistance of the EBR bio-resin film. Although the values of the swelling degree of the films were not statistically different, it can be noted that the swelling degree of the EBR film decreased with increasing epoxy bio-resin contents in the films from EBR (10:2.5) to EBR (10:10). The EBR (10:10) film had the lowest swelling degree, 2.9%, as compared to the EBR (10:2.5) film, which had a swelling degree of 2.29%. This phenomenon of decreased swelling degree of the resin film is related to the fact that EBR (10:10) had a stronger interaction with the acrylic acid, which inhibited water penetration into the film network and altered the swelling degree of the EBR film. In a similar manner, the water solubility of the EBR films was measured, and it was found that the values of water solubility for the films ranged from 0.76 to 0.29%. The fact that the water solubility values of the films did not differ significantly showed that the EBR films have water-resistant properties. Although EBR films were not soluble enough in water, they could potentially lead to acid hydrolysis of the resin during the curing process [1]. This phenomenon might be associated with the heterogeneous structure of the EBR film, which reduces the cohesiveness of the resin matrix and promotes water permeation [34,35].

## 4. Conclusions

The epoxidation reaction of soybean oil was conducted using different equivalent weights of formic acid as an oxidizing agent. The study was designed to investigate the impact of formic acid as an oxidant on the epoxidation of soybean oil and the subsequent formulation of bio-based epoxy resins using the resulting epoxidized soybean oil. The successful synthesis of epoxidized soybean oil with varying formic acid concentrations was confirmed by evaluating the epoxy yields as well as thermal and mechanical properties. The results revealed that higher formic acid concentrations, particularly at a ratio of 10:10, yielded high selectivity (93.68%) and conversion (88.80%) simultaneously under mild reaction conditions. The resulting resin film, EBR (10:10), exhibited a superior transparency, tensile strength, and swelling degree compared to the EBR films produced using a 10:2.5 ratio. EBR (10:10) demonstrated the most favorable characteristics among all the samples examined. In addition, the EBR (10:2.5) film did not significantly enhance the water resistance and mechanical properties of the samples, due to incompatibility and acid hydrolysis. Furthermore, ESO, which was epoxidized using a 10:1 ratio of soybean oil and formic acid in the presence of a green catalyst, did not undergo epoxidation and did not form any resin during the reactions.

## Figures and Tables

**Table 1:** Opacity (mm) and water resistance of the epoxidized bio-resin films (EBRs).

Samples	Opacity (mm^−1^)	Swelling Degree (%)	Water Solubility (%)
EBR (10:1)	NA	NA	NA
EBR (10:2.5)	0.122 ± 0.002	2.291 ± 0.46	0.762 ± 0.21
EBR (10:5)	0.172 ± 0.003	2.053 ± 0.01	0.402 ± 0.02
EBR (10:7.5)	0.170 ± 0.008	2.45 ± 0.32	0.354 ± 0.03
EBR (10:10)	0.229 ± 0.010	2.905 ± 0.47	0.294 ± 0.12

NA: not applicable.

## Data Availability

Data will be made available on request.
